# Prognostic significance of inflammation-related and electrolyte laboratory variables in patients with malignant pleural mesothelioma

**DOI:** 10.3389/fmed.2023.1099685

**Published:** 2023-04-05

**Authors:** Yuan Zhang, Jie Li, Shu Zhang

**Affiliations:** Department of Respiratory and Critical Care Medicine, Beijing Institute of Respiratory Medicine and Beijing Chao Yang Hospital, Capital Medical University, Beijing, China

**Keywords:** malignant pleural mesothelioma, prognosis, calcium, platelet-to-lymphocyte ratio, monocyte-to-white blood cell ratio

## Abstract

**Objective:**

Malignant pleural mesothelioma (MPM) is a kind of pleural cancer characterized by low incidence but high invasiveness. There is heterogeneity in survival among patients with MPM. Inflammation-related and electrolyte laboratory variables were previously reported as potential predictors of survival. We evaluated the relationship between overall survival and pre-treatment biomarkers.

**Materials and methods:**

Patients diagnosed with MPM in Beijing Chaoyang Hospital for more than 10 years were screened for this study. All basic, clinical, radiologic and laboratory variables were collected. The COX univariable and multivariable analysis were used to explore prognostic related risk factors.

**Results:**

Ninety patients with MPM were included. The median follow-up of all patients was 57 months [interquartile range (IQR): 27–100 months]. The median survival time was 24 months (IQR: 12–52 months). Univariate survival analyses indicated that age, Eastern Cooperative Oncology Group Performance Status, treatment, erythrocyte sedimentation rate, calcium, lymphocyte, hemoglobin, platelet-to-lymphocyte ratio (PLR), and monocyte-to-white blood cell ratio (MWR) were significantly related to survival. Multivariable analysis demonstrated that age [hazard ratio (HR), 2.548; 95% confidence interval (CI) 1.145–5.666; *p* = 0.022], calcium (HR, 0.480; 95% CI 0.270–0.855; *p* = 0.013), PLR (HR, 2.152; 95% CI 1.163–3.981; *p* = 0.015), and MWR (HR, 3.360; 95% CI 1.830–6.170; *p* < 0.001) might have a significant impact on the prognosis.

**Conclusion:**

Calcium, MWR, and PLR might be related to the prognosis of MPM patients. Analyzing the relationship between the results of inflammation-related and electrolyte laboratory variables in peripheral blood and prognosis could help clinicians evaluate the situation of patients.

## 1. Introduction

Malignant pleural mesothelioma (MPM) is a specific cancer of the pleura. Although its occurrence is very rare, its incidence is also increasing all over the world. It is generally considered to be related to asbestos exposure (1). Overall, the prognosis of MPM patients is poor, but there are great individual differences between patients (2). The main reason for the difference in prognosis is that different patients have different risk factors. Therefore, it is very important to determine the prognostic risk factors of MPM. Through the comprehensive analysis of the risk factors of patients, the prognosis of patients can be judged to a certain extent. For those patients whose prognoses are likely to be better, more effective treatment should be selected. For patients with potentially poor prognoses, clinicians should choose relatively conservative therapy to ensure the quality of life of patients (3).

It is well known that the pathogenesis of MPM is closely correlated with occupational or environmental exposure to asbestos fibers. After the patient inhales slender asbestos fibers, asbestos will stimulate the pleura and be deposited locally (4, 5). MPM patients may respond to stimuli, release cytokines, produce a systemic inflammatory response, together with other pathological pathways, eventually lead to immunosuppression, and then lead to adverse prognosis (6–8).

Extensive studies have examined prognostic factors in MPM, including basic epidemiological variables, laboratory examination results, imaging features, and pathological characteristics (9). For clinicians, it is very basic and important to regularly detect the results of patients’ peripheral blood. Therefore, it has important clinical significance to judge whether the blood test results can be used as a risk factor for prognosis. As systemic inflammatory response indicators, neutrophils, lymphocytes (10), neutrophil-to-lymphocyte ratio (NLR) (7, 11), monocytes (12), platelets (13, 14), platelet-to-lymphocyte ratio (PLR) (15), and other markers (16) are potentially associated with the prognosis of MPM. Several studies suggest that electrolytes, such as calcium and potassium (17), may also regulate tumor cell migration, further affecting cancer progression. However, these results remain controversial (18), and currently, the inflammatory-related features that predict survival have not been accurately determined. This study aimed to identify which inflammation-related laboratory variables and electrolyte markers were significantly correlated with prognosis.

## 2. Materials and methods

### 2.1. Study population

This study firstly collected patients who were diagnosed as MPM in Beijing Chaoyang Hospital from June 1, 2010 to July 1, 2021. Inclusion criteria were pathologically confirmed MPM patients who had not undergone surgery. Exclusion criteria were missing information or patients with autoimmune disease. Patients were screened for research according to the above criteria, and all of the clinical and pathological information was collected comprehensively. All patients had signed written informed consent.

### 2.2. Data collection

Five aspects of patient information at the time of diagnosis through the electronic medical record system were collected. The first part is the basic information of patients, including age, sex, smoking history, history of asbestos, and Eastern Cooperative Oncology Group Performance Status (ECOG PS). The second part is disease-related factors, including diagnostic methods and tumor histology. The third part is the treatment-related factors, including surgery, chemotherapy and anti-angiogenesis therapy. Surgery included pleurectomy/decortication and extra-pleural pneumonectomy. The chemotherapy regimen is mainly pemetrexed combined with platinum. The anti-angiogenic drug is mainly bevacizumab. The fourth part is the radiological features including tumor location, interlobar fissure pleural invasion， mediastinal pleural invasion, mediastinal lymph node invasion，thoracostenosis and pleural calcification. The fifth part is the laboratory variables, including the count of white blood cell (WBC), neutrophil, lymphocyte, monocyte, eosinophil, basophil, erythrocyte, hemoglobin, platelet, erythrocyte sedimentation rate (ESR), sodium, potassium, and calcium. The sixth part is the blood cell ratio. The monocyte to leukocyte ratio (MWR) is the ratio of monocyte count to WBC count. PLR, NLR, the platelet-to-white blood cell ratio (PWR) and the eosinophil-to-lymphocyte ratio (ELR) are calculated in the same way.

### 2.3. Screening and follow-up strategies

In order to evaluate tumor progression and therapeutic efficacy, all patients with MPM diagnosed in our center are required to have peripheral blood examination and chest computed tomography (CT) at least every half a year within 2–3 years after diagnosis and at least every year thereafter. Collect all follow-up examination information from the diagnosis of each patient in the electronic medical system to December 31, 2021. Finally, all patients were followed up by telephone on December 31, 2021, so as to know the treatment and current survival of patients in other hospitals.

### 2.4. Statistical analysis

Continuous variables that were not normally distributed are represented by the median and interquartile range (IQR). Other continuous variables were shown as the mean ± standard deviation (SD). The optimal cut-off values for continuous variables were calculated according to the receiver operating characteristic (ROC) curves. Frequencies and percentages characterized categorical data. The survival curve was drawn by Kaplan–Meier, and the difference between the two groups was tested by log-rank. Univariate and multivariate analyses were analyzed by Cox regression analysis. Multivariate analysis included two parts: the first part was a statistically significant univariate, and the second part was the recognized basic independent variables related to survival, especially age, gender and histological subtype. Other analyses were performed by SPSS software (version 25.0; IBM, Armonk, NY, USA). Professional epidemiologists reviewed the statistical methods in this paper.

## 3. Results

### 3.1. Patient characteristics

This study collected 101 patients diagnosed with MPM in our hospital within 10 years. Eleven patient was excluded because of incomplete baseline and radiological data. Finally, 90 eligible patients were included. Sixty-three patients (70.0%) died during the follow-up period. Of the 90 patients, 48 (53.3%) were male. The overall median age was 65.0 years, and 24 (26.7%) patients had been exposed to asbestos. Thirty-nine (43.3%) patients had a history of smoking. There were 38 patients with hypertension, 21 patients with diabetes, 6 patients with coronary heart disease, 1 patient with COPD, 1 patient with asthma, and 2 patients with pulmonary tuberculosis. In order to diagnosis, 3 (3.3%) patients had pleural effusion aspirated for cellular wax block, 16 (17.8%) patients had ultrasound-guided pleural biopsy, 61 (67.8%) patients were diagnosed by medical thoracoscopy, ten (11.1%) patients underwent video-assisted thoracoscopic surgery (VATS). This MPM cohort included non-epithelioid subtype (34.4%; *n* = 31), including sarcomatoid, biphasic, and undefined types, the others were epithelioid subtype (65.6%; *n* = 59). When diagnosed, the number and proportion of patients with an ECOG PS of 2 or 3 were 20 (22.2%). Eight (8.9%) patients with relatively poor ECOG PS received supportive care. The other patients received chemotherapy alone or in combination with antiangiogenic therapy. In detail, most patients received pemetrexed combined with platinum ± bevacizumab (Table 1).

**Table 1:** Baseline characteristics and laboratory variables of the study population.

Characteristic	Total (*N* = 90)	Survival (*n* = 27)	Death (*n* = 63)
*Age^1^*	65.0 (58.0, 72.0)	65.0 (55.0, 70.0)	65.0 (58.0, 74.0)
*Gender*			
Male	48 (53.3)	12 (44.4)	36 (57.1)
Female	42 (46.7)	15 (55.6)	27 (42.9)
*Smoke history*			
Never	51 (56.7)	16 (59.3)	35 (55.6)
Current and former	39 (43.3)	11 (40.7)	28 (44.4)
*Asbestos exposure*			
No	66 (73.3)	22 (81.5)	44 (69.8)
Yes	24 (26.7)	5 (18.5)	19 (30.2)
*Hypertension*			
No	52 (57.8)	14 (51.9)	38 (60.3)
Yes	38 (42.2)	13 (48.1)	25 (39.7)
Diabetes mellitus			
No	69 (76.7)	19 (70.4)	50 (79.4)
Yes	21 (23.3)	8 (29.6)	13 (20.6)
*Coronary artery heart disease*			
No	84 (93.3)	25 (92.6)	59 (93.7)
Yes	6 (6.7)	2 (7.4)	4 (6.3)
*ECOG PS*			
0–1	70 (77.8)	24 (88.9)	46 (73.0)
2–3	20 (22.2)	3 (11.1)	17 (27.0)
*Diagnostic methods*			
Cell blocks from malignant pleural effusion	3 (3.3)	2 (7.4)	1 (1.6)
Ultrasound-guided percutaneous biopsy	16 (17.8)	3 (11.1)	13 (20.6)
Video-Assisted Thoracic Surgery	10 (11.1)	6 (22.2)	4 (6.3)
Medical thoracoscopy	61 (67.8)	16 (59.3)	45 (71.4)
*Histology*			
Epithelioid	59 (65.6)	19 (70.4)	40 (63.5)
Non-epithelioid	31 (34.4)	8 (29.6)	23 (36.5)
*Treatment*			
Best supportive care	8 (8.9)	0 (0)	8 (12.7)
Chemotherapy ± anti-angiogenesis therapy	82 (91.1)	27 (100)	55 (87.3)
*Tumor location*			
Unilateral	82 (91.1)	25 (92.6)	57 (90.5)
Bilateral	8 (8.9)	2 (7.4)	6 (9.5)
*Pleural invasion of interlobar fissure*			
No	55 (61.1)	18 (66.7)	37 (61.1)
Yes	35 (38.9)	9 (33.3)	35 (38.9)
*Mediastinal pleural invasion*			
No	69 (76.7)	19 (70.4)	50 (79.4)
Yes	21 (23.3)	8 (29.6)	13 (20.6)
*Mediastinal lymph node invasion*			
No	72 (80.0)	23 (85.2)	49 (77.8)
Yes	18 (20.0)	4 (14.8)	14 (22.2)
*Thoracostenosis*			
No	67 (74.4)	17 (63.0)	50 (79.4)
Yes	23 (25.6)	10 (37.0)	13 (20.6)
*Pleural calcification*			
No	71 (78.9)	23 (85.2)	48 (76.2)
Yes	19 (21.1)	4 (14.8)	15 (23.8)
ESR^2^	15.0 (7.8, 25.8)	12.0 (5.0, 18.0)	15.0 (8.0, 38.0)
Sodium^3^	141.2 (139.4, 142.7)	141.4 (140.1, 142.6)	141.2 (138.5, 142.8)
Potassium^3^	4.0 (3.7, 4.2)	4.1 (3.7, 4.3)	3.9 (3.6, 4.2)
Calcium^3^	2.2 (2.1, 2.3)	2.2 (2.2, 2.3)	2.1 (2.1, 2.2)
WBC^4^	6.4 (5.0, 8.1)	6.0 (4.8, 7.5)	6.5 (5.1, 8.6)
Neutrophil^4^	4.2 (3.4, 5.7)	3.8 (2.9, 4.8)	4.3 (3.5, 6.2)
Lymphocyte^4^	1.6 (1.1, 1.9)	1.6 (1.3, 2.0)	1.5 (1.0, 1.9)
Monocyte^4^	0.4 (0.3, 0.6)	0.4 (0.3,0.5)	0.5 (0.4, 0.7)
Eosinophil^4^	0.110 (0.060, 0.170)	0.140 (0.090, 0.180)	0.100 (0.060, 0.170)
Basophil^4^	0.020 (0.010, 0.030)	0.020 (0.020, 0.060)	0.020 (0.010, 0.030)
Erythrocyte^4^	4.5 (4.1, 4.8)	4.6 (4.3, 4.8)	4.4 (4.0, 4.8)
Hemoglobin^5^	134.0 (121.0, 145.3)	140.0 (130.0, 150.0)	131.0 (117.0, 142.0)
Platelet^4^	242.0 (188.3, 289.3)	227.0 (189.0, 266.0)	248.0 (185.0, 328.0)
PLR	169.3 (126.3, 229.0)	144.2 (104.6, 179.5)	182.0 (140.3, 283.9)
MWR	0.068 (0.057, 0.081)	0.067 (0.050, 0.076)	0.068 (0.057, 0.088)
NLR	2.8 (2.0, 4.4)	2.3 (1.7, 3.2)	3.2 (2.2, 4.9)
PWR	36.0 (29.7, 50.2)	35.2 (31.1, 50.1)	36.8 (28.9, 50.4)
ELR	0.072 (0.042, 0.117)	0.094 (0.059, 0.111)	0.067 (0.039, 0.130)

SD, standard deviation; ECOG PS, Eastern Cooperative Oncology Group Performance Status; ESR, erythrocyte sedimentation rate; IQR, interquartile range; WBC, white blood cell; PLR, platelet-to-lymphocyte ratio; MWR, monocyte-to-white blood cell ratio; NLR, neutrophil-to-lymphocyte ratio; PWR, platelet-to-white blood cell ratio; ELR, eosinophil-to-lymphocyte ratio.

Units: ^1^years, ^2^mm/h, ^3^mmol/L, ^4^10^9^/L, ^5^g/L.

Eighty-two patients (91.1%) only invaded unilateral pleura. 35 (38.9%) patients had interlobar fissure pleural invasion. 21 (23.3%) patients had mediastinal pleural invasion. 18 (20.0%) patients had mediastinal lymph node invasion. 23 (25.6%) patients had thoracostenosis. 19 (21.1%) patients had pleural calcification (Table 1).

### 3.2. Cut-off values for laboratory variables at diagnosis for survival analysis

**Figure 1:**
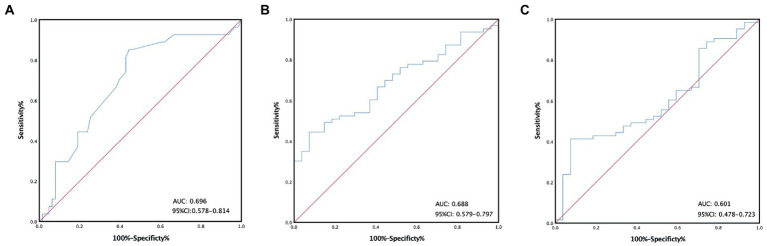
Receiver operating characteristic curves stratified by the different calcium **(A)**, platelet-to-lymphocyte ratio **(B)**, monocyte-to-white ratio **(C)** groups in patients with malignant pleural mesothelioma.

All of the hematological parameters and blood cell ratio were expressed as the median and IQR (Table 1). According to the ROC curve, we calculated cutoff values for all laboratory parameters, such as the cutoff value of calcium, which was 2.1(AUC: 0.696, 95%CI: 0.578–0.814) (Figure 1A). All patients were divided into two groups according to the cutoff value. There were 39 patients (43.3%) in the low-calcium group (calcium≤2.1). There were 51 patients (56.7%) in the high-calcium group (calcium>2.1). The cutoff value of PLR is 199.2 (AUC: 0.688, 95%CI: 0.579–0.797) (Figure 1B). There were 60 patients (66.7%) in the low-PLR group (PLR ≤ 199.2). There were 30 patients (33.3%) in the high-PLR group (PLR > 199.2). The cutoff value of MWR is 0.079 (AUC: 0.601, 95%CI: 0.478–0.723) (Figure 1C). There were 62 patients (68.9%) in the low-MWR group (MWR ≤ 0.079). There were 28 patients (31.1%) in the high-MWR group (MWR > 0.079). Other laboratory indicators were calculated in the same way (Table 2).

**Table 2:** Univariable Cox regression analyses between baseline characteristics, laboratory variables, and OS of malignant pleural mesothelioma patients.

Characteristic	No.(%)	HR	95%CI	*p* value
*Age^1^*				
≤56.5	19 (21.1)	1		
>56.5	71 (78.9)	2.058	1.037–4.083	0.039
*Gender*				
Male	48 (53.3)	1		
Female	42 (46.7)	0.805	0.485–1.337	0.402
Smoke history				
Never	51 (56.7)	1		
Current and former	39 (43.3)	1.251	0.754–2.074	0.386
*Asbestos exposure*				
No	66 (73.3)	1		
Yes	24 (26.7)	1.291	0.748–2.226	0.359
Hypertension				
No	52 (57.8)	1		
Yes	38 (42.2)	0.757	0.453–1.266	0.288
*Diabetes mellitus*				
No	69 (76.7)	1		
Yes	21 (23.3)	0.992	0.538–1.832	0.981
*Coronary artery heart disease*				
No	84 (93.3)	1		
Yes	6 (6.7)	1.316	0.474–3.654	0.598
*ECOG PS*				
0–1	70 (77.8)	1		
2–3	20 (22.2)	2.110	1.184–3.761	0.011
*Diagnostic methods*				
Cell blocks from malignant pleural effusion	3 (3.3)	1		0.420
Ultrasound-guided percutaneous biopsy	16 (17.8)	1.440	0.185–11.236	0.728
Video-assisted thoracic surgery	10 (11.1)	0.587	0.065–5.322	0.636
Medical thoracoscopy	61 (67.8)	0.944	0.128–6.986	0.955
*Histology*				
Epithelioid	59 (65.6)	1.251	0.742–2.109	0.400
Non-epithelioid	31 (34.4)			
*Treatment*				
Best supportive care	8 (8.9)	1		
Only Chemotherapy ± anti-angiogenesis therapy	82 (91.1)	0.403	0.188–0.864	0.020
Tumor location				
Unilateral	82 (91.1)	1		
Bilateral	8 (8.9)	1.186	0.509–2.761	0.693
*Interlobar fissure pleural invasion*				
No	55 (61.1)	1		
Yes	35 (38.9)	1.220	0.736–2.024	0.440
*Mediastinal pleural invasion*				
No	69 (76.7)			
Yes	21 (23.3)	1		
Mediastinal lymph node invasion		0.811	0.439–1.497	0.503
No	72 (80.0)	1		
Yes	18 (20.0)	1.678	0.919–3.062	0.092
*Thoracostenosis*				
No	67 (74.4)	1		
Yes	23 (25.6)	0.727	0.394–1.342	0.308
*Pleural calcification*				
No	71 (78.9)	1		
Yes	19 (21.1)	1.256	0.701–2.250	0.444
*ESR^2^*				
≤20.5	60 (66.7)	1		
> 20.5	30 (33.3)	1.852	1.112–3.084	0.018
Sodium^3^				
≤143.3	73 (81.1)	1		
>143.3	17 (18.9)	1.096	0.603–1.992	0.764
*Potassium^3^*				
≤4.0	53 (58.9)	1		
>4.0	37 (41.1)	0.744	0.437–1.268	0.277
*Calcium^3^*				
≤2.1	39 (43.3)	1		
>2.1	51 (56.7)	0.526	0.318–0.870	0.012
*WBC^4^*				
≤8.0	65 (72.2)	1		
>8.0	25 (27.8)	1.206	0.705–2.063	0.494
*Neutrophil^4^*				
≤5.9	70 (77.8)	1		
>5.9	20 (22.2)	1.266	0.712–2.253	0.421
*Lymphocyte^4^*				
≤1.1	22 (24.4)	1		
>1.1	68 (75.6)	0.439	0.255–0.756	0.003
*Monocyte^4^*				
≤0.5	64 (70.0)	1		
>0.5	27 (30.0)	1.666	0.993–2.793	0.053
*Eosinophil^4^*				
≤0.095	38 (42.2)	1		
>0.095	52 (57.8)	0.823	0.499–1.356	0.444
*Basophil^4^*				
≤0.015	31 (34.4)	1		
>0.015	59 (65.6)	0.699	0.420–1.163	0.168
*Erythrocyte^4^*				
≤4.2	31 (34.4)	1		
>4.2	59 (65.6)	0.670	0.404–1.111	0.121
*Hemoglobin^5^*				
≤124.5	27 (30.0)	1		
>124.5	63 (70.0)	0.570	0.340–0.955	0.033
*Platelet^4^*				
≤289.5	68 (75.6)	1		
>289.5	22 (24.4)	1.471	0.839–2.581	0.178
*PLR*				
≤199.2	60 (66.7)	1		
>199.2	30 (33.3)	2.155	1.294–3.589	0.003
*MWR*				
≤0.079	62 (68.9)	1		
>0.079	28 (31.1)	3.633	2.097–6.296	<0.001
*NLR*				
≤3.7	58 (64.4)	1		
>3.7	32 (35.6)	1.396	0.833–2.341	0.206
*PWR*				
≤27.7	17 (18.9)	1		
>27.7	73 (81.1)	0.697	0.383–1.268	0.238
*ELR*				
≤0.055	32 (35.6)	1		
>0.055	58 (64.4)	0.824	0.495–1.373	0.458

HR, hazard ratio; CI, confidence interval; ECOG PS, Eastern Cooperative Oncology Group Performance Status; ESR, erythrocyte sedimentation rate; IQR, interquartile range; SD, standard deviation; WBC, white blood cell; PLR, platelet-to-lymphocyte ratio; MWR, monocyte-to-white blood cell ratio; NLR, neutrophil-to-lymphocyte ratio; PWR, platelet-to-white blood cell ratio; ELR, eosinophil-to-lymphocyte ratio.

Units: ^1^years, ^2^mm/h, ^3^mmol/L, ^4^10^9^/L, ^5^g/L.

### 3.3. Survival analysis

**Figure 2:**
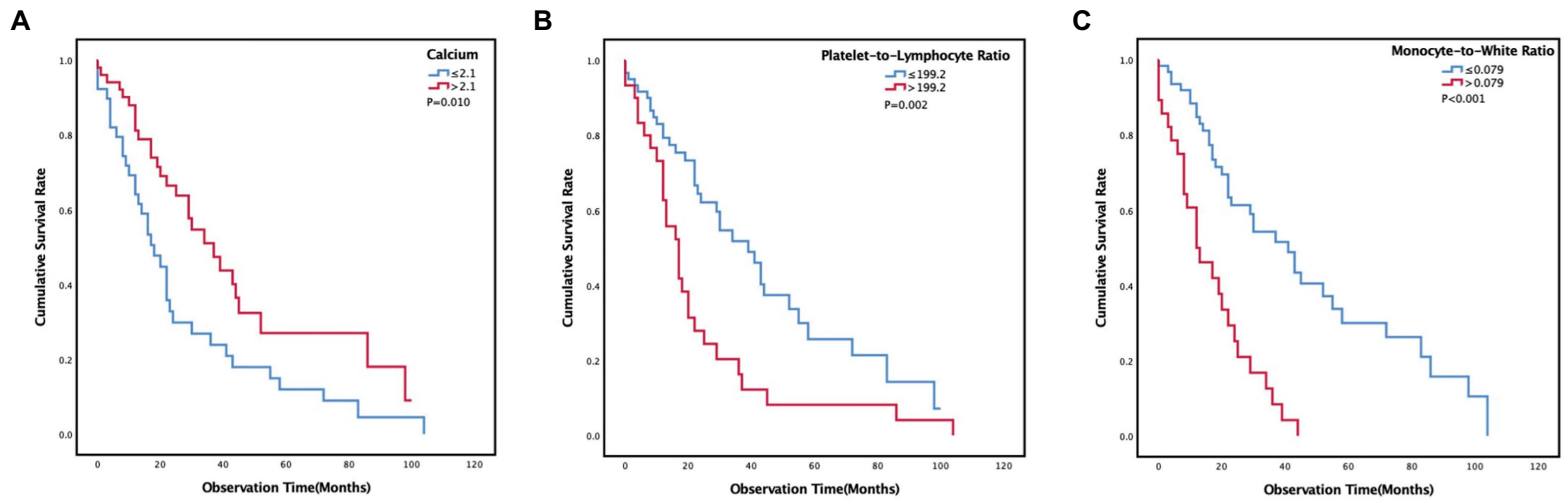
Kaplan–Meier curves estimates of prognosis in different calcium **(A)**, platelet-to-lymphocyte ratio **(B)**, monocyte-to-white ratio **(C)** groups of malignant pleural mesothelioma patients.

The median OS was 24 (12, 52) months. The survival rates of 6 months, 1 year, 2 years, and 3 years were 87, 66, 39, and 24%. Univariate survival analyses indicated that age (*p* = 0.039), ECOG PS (*p* = 0.011), treatment (*p* = 0.020), erythrocyte sedimentation rate (*p* = 0.018), calcium (*p* = 0.012), lymphocyte (*p* = 0.003), hemoglobin (*p* = 0.033), PLR (p = 0.003) and MWR (*p* < 0.001) might be significantly associated with survival (Table 2). Patients in the high-calcium group had a better prognosis than those in the low-calcium group (*p* = 0.010) (Figure 2A). Compared with the low PLR group and the low MWR group, the high LWR group (*p* = 0.002) (Figure 2B) and the high MWR group (*p* < 0.001) (Figure 2C) had a worse prognosis. There was no significant correlation between other independent variables and survival (Table 2). Multivariate analyses showed that age [hazard ratio (HR), 2.548; 95% confidence interval (CI) 1.145–5.666; *p* = 0.022], calcium (HR, 0.480; 95% CI 0.270–0.855; *p* = 0.013), PLR (HR, 2.152; 95% CI 1.163–3.981; *p* = 0.015), and MWR (HR, 3.360; 95% CI 1.830–6.170; p < 0.001) were considered as the possible prognostic factors for MPM patients (Table 3).

**Table 3:** Multivariable Cox regression analyses between baseline characteristics, laboratory variables, and OS of malignant pleural mesothelioma patients.

Characteristic	HR	95%CI	P value
*Age^1^*			
≤56.5	1		
>56.5	2.548	1.145–5.666	0.022
*Gender*			
Male	1		
Female	0.766	0.427–1.375	0.372
*ECOG PS*			
0–1	1		
2–3	1.609	0.692–3.737	0.269
Histology			
Epithelioid	1		
Non-epithelioid	0.840	0.466–1.516	0.563
Treatment			
Best supportive care	1		
Chemotherapy ± anti-angiogenesis therapy	0.694	0.233–2.067	0.512
ESR^2^			
≤20.5	1		
>20.5	1.256	0.695–2.269	0.451
Calcium^3^			
≤2.1	1		
>2.1	0.480	0.270–0.855	0.013
Hemoglobin^4^			
≤124.5	1		
>124.5	0.965	0.503–1.854	0.916
PLR			
≤199.2	1		
>199.2	2.152	1.163–3.981	0.015
*MWR*			
≤0.079	1		
>0.079	3.360	1.830–6.170	<0.001

HR, hazard ratio; CI, confidence interval; ESR, erythrocyte sedimentation rate; PLR, platelet-to-lymphocyte ratio; MWR, monocyte-to-white blood cell ratio.

Units: ^1^years, ^2^mm/h, ^3^mmol/L, ^4^g/L.

## 4. Discussion

The overall prognosis of MPM patients is poor, but there are significant individual differences in patient prognosis. Therefore, identifying major predictors of OS in MPM patients is essential. However, the results of existing literature were inconsistent, and the predictors in these studies were diverse in several ways. In this context, the current study analyzed the prognosis of MPM patients and its major determinants, focusing on inflammation-related and electrolyte laboratory variables.

### 4.1. Calcium and prognosis

Interestingly, our study found a significant correlation between low calcium and poorer patient survival. At present, the research on the role of calcium in mesothelioma is insufficient. KCa1.1 (17) and T-type Ca2+ channels (19) highly expressed in patient tumor specimens were found to regulate cancer proliferation and development. However, the comprehensive and detailed mechanism by which calcium affects the prognosis of patients with pleural mesothelioma remains to be further elucidated.

### 4.2. PLR and prognosis

Previous studies have shown that systemic inflammatory responses in cancer patients may lead to poor prognosis. Pretreatment peripheral blood laboratory tests in patients with MPM at the time of diagnosis may reflect the original inflammatory status (15). Elevated platelet counts may reflect systemic inflammation, and lymphocyte counts may be associated with immunosuppression. PLR that combines inflammatory and immunosuppressive indicator status may be better biomarker than platelets and lymphocytes alone. In this study, we found a significant correlation between the PLR and prognosis in patients with MPM, consistent with previous studies (15, 20). There are several possible explanations for this finding. First, previous studies have confirmed that platelets accumulate and produce factors in tumor capillaries, protect tumor cells from destruction, increase the blood supply of tumor cells, and ultimately promote cancer progression (21). Furthermore, lymphocytes interact with a variety of cytokines, which ultimately affect the growth and metastasis of tumor cells (22). Moreover, the number of lymphocytes in patients with MPM affects the efficacy of chemotherapy and immunotherapy to a certain extent (23, 24). Therefore, lymphocytes are related to the subtype and prognosis of MPM (25, 26). In summary, the PLR is closely associated with patient prognosis.

### 4.3. MWR and prognosis

This study suggests that MWR may be related to the prognosis of MPM patients to some extent. The prognostic role of MWR has been studied in several tumors other than MPM. Previous studies suggest that elevated monocyte counts may contribute to poor prognosis in MPM patients (12) and other solid tumors, such as gastric cancer (27), pancreatic ductal adenocarcinoma (28), lung cancer (29), and prostate cancer (30). Based on the above studies, it is reasonable to believe that MWR may be a prognostic factor for survival in MPM patients.

First, monocytes perform various functions that contribute to pro-tumoral immunity, including phagocytosis, tumoricidal mediator secretion, angiogenesis, extracellular matrix remodeling and lymphocyte aggregation during cancer development (31). Second, high peripheral blood mononuclear cell count is also a manifestation of a high tumor burden, which can reflect increased macrophage counts in tissues and organs (32). Tumor-associated macrophages can secrete a variety of cytokines and participate in tumor progression leading to enhanced tumor cell proliferation, angiogenesis, and immunosuppression and subsequently supporting invasion and metastasis (33). These results suggest that it is plausible that increased MWR may lead to poor prognosis in MPM patients.

### 4.4. Age and prognosis

Our study reported that age was significantly related to prognosis. In general, younger patients have lower ECOG PS scores, fewer underlying diseases, and more opportunities to receive chemotherapy and anti-angiogenesis therapy. As a result, their prognosis may be better, consistent with a previous study (34, 35). However, whether age affect the prognosis of patients is still controversial (36, 37). This result should be validated in subsequent large-sample studies.

### 4.5. Limitations

There are still many limitations in this retrospective study. First, it was conducted in a single-center, so few patients were enrolled. The gap between groups of some factors is not balanced. Due to the nature of the study, bias is unavoidable. Second, the population included in this study were non-surgical MPM patients who had undergone chemotherapy. The relatively single population may make the results one-sided and not applicable to all MPM patients. Additionally, some factors were difficult to assess and not analyzed in detail, so not all prognostic risk factors were included. For example, due to the rarity of patients with mesothelioma, this study took a long time, and some patients failed to undergo systemic examination. Therefore, most of the patients could not been carried out accurate clinicopathological staging.

## 5. Conclusion

In conclusion, our data show that calcium, MWR, and PLR are correlated with MPM prognosis. Additional large sample prospective studies are urgently required to validate the results.

## Data availability statement

The original contributions presented in the study are included in the article/supplementary material, further inquiries can be directed to the corresponding authors.

## Ethics statement

The studies involving human participants were reviewed and approved by the Ethics Committees of the Beijing Chao-Yang Hospital, Capital Medical University. The patients/participants provided their written informed consent to participate in this study.

## Author contributions

YZ: conceptualization, investigation, data curation, formal analysis, and writing—original draft. SZ and JL: conceptualization, methodology, resources, supervision, and writing—review and editing. All authors have read and approved the manuscript.

## Conflict of interest

The authors declare that the research was conducted in the absence of any commercial or financial relationships that could be construed as a potential conflict of interest.

## Publisher’s note

All claims expressed in this article are solely those of the authors and do not necessarily represent those of their affiliated organizations, or those of the publisher, the editors and the reviewers. Any product that may be evaluated in this article, or claim that may be made by its manufacturer, is not guaranteed or endorsed by the publisher.
